# New species of *Paullinia* (Sapindaceae) from continental tropical America

**DOI:** 10.3897/phytokeys.114.29351

**Published:** 2018-12-31

**Authors:** Pedro Acevedo-Rodríguez, Genise Vieira Somner

**Affiliations:** 1 Department of Botany, National Museum of Natural History, Smithsonian Institution, P.O. Box 37012, Washington, D.C. 20013-7012, USA National Museum of Natural History, Smithsonian Institution Washington United States of America; 2 Universidade Federal Rural do Rio de Janeiro, Departamento de Botânica, Caixa postal 74582, CEP: 23851-970 – Seropédica, Rio de Janeiro, Brazil Universidade Federal Rural do Rio de Janeiro Rio de Janeiro Brazil

**Keywords:** Neotropics, Sapindales, Paullinieae, lianas, climbing shrubs, Amazonia, Brazil, Ecuador, Guatemala, Honduras, Peru

## Abstract

Six new species are described in the large Neotropical genus *Paullinia* (Sapindaceae), *P.cidii*, *P.decorticans*, *P.fruticosa*, *P.hondurensis*; *P.martinellii* and *P.wurdackii*. In addition, they are illustrated and contrasted to the morphologically most similar species currently known. The new species were discovered while working on a forthcoming revision of the genus.

## Abstract (pt)

Ao trabalhar na revisão do grande gênero neotropical *Paullinia*, descobrimos várias espécies novas para a ciência, dentre estas, seis são descritas *P.cidii*, *P.decorticans*, *P.fruticosa*, *P.hondurensis*, *P.martinellii* e *P.wurdackii*. Além disso, estas são ilustradas e contrastadas com outras espécies conhecidas atualmente mais morfologicamente semelhantes.

## Introduction

While working on a revision of the Neotropical genus *Paullinia*, we discovered several species new to science. *Paullinia* is one of the largest genera of Sapindaceae with more than 200 species. A recent taxonomic paper establishes *Paullinia* as monophyletic and related to other genera of the Neotropical tribe Paullinieae (Acevedo-Rodríguez et al. 2017). These species although not yet placed in a phylogenetic system, are sufficiently distinct as to be considered new to science. These are here described and contrasted to species considered their closest relatives due to their overall morphological similarities.

## Materials and methods

The descriptions of the new species are based on herbarium collections, field notes and photographs taken by the senior author. The recognition of the new species is based on comparative morphological studies that have shown consistent morphological uniqueness (two or more characters) correlated with a particular geographical area and or habitat. Descriptions of the new species follow the format used for the family in our previous publications, ongoing floristic treatments and monographic studies of the genus (Acevedo-Rodríguez and Somner in prep.). Herbarium acronyms given for the studied collections follow Index Herbariorum (Thiers continuously updated). Preliminary conservation status of the new species was assessed by using IUCN guidelines (2001).

## Taxonomic treatment

### Paullinia cidii

Taxon classificationPlantaeSapindalesSapindaceae

Somner & Acev.-Rodr. sp. nov.

urn:lsid:ipni.org:names:77192860-1

Fig. 1

#### Diagnosis.

*Paulliniacidii* differs from *P.bipinnata* Poir., *P.filicifolia* Cuatrec. and *P.hondurensis* Acev.-Rodr. & Somner, the only three congeners with partially tripinnate leaves with triangular outline, by its globose, long-stipitate, unwinged capsules (vs. shortly stipitate, and winged). It is unique within *Paullinia* by having the referred combination of characters.

**Figure 1.**
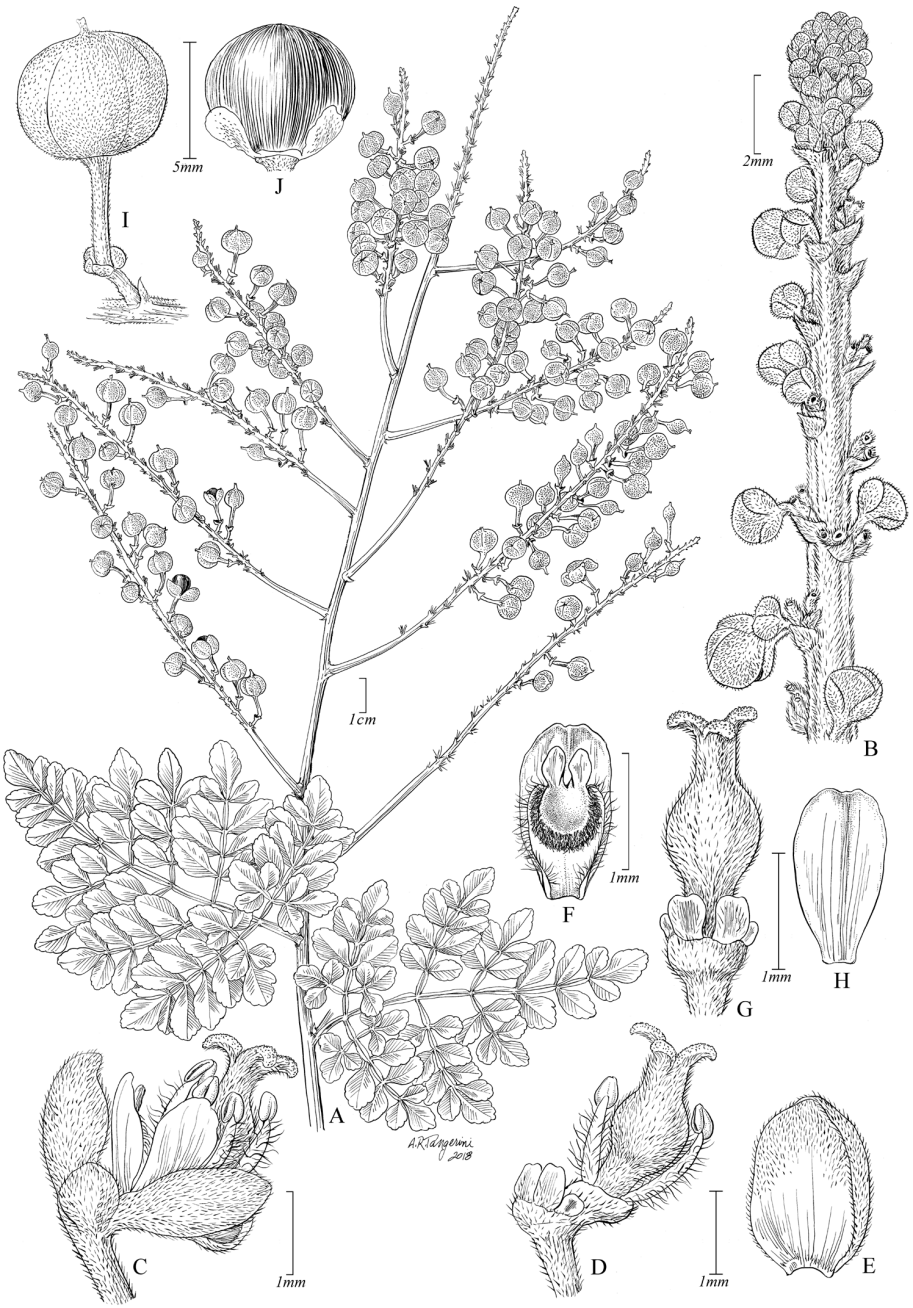
*Paulliniacidii*. **A** Fruiting branch **B** Detail of inflorescence with flower buds **C** Pistillate flower, lateral view **D** Pistillate flower with removed perianth, showing nectary glands and torus **E** Posterior sepal, frontal view **F** Posterior petal with appendix, frontal view **G** Gynoecium and nectary glands at base, posterior view **H** Posterior petal dorsal view **I** Mature capsule **J** Seed, dorsal view. **A, I, J** from *J.E.L.S. Ribeiro et al 2660* (US) **B–H** from *Zartman 7124* (US).

#### Type.

BRAZIL. Amazonas; Mun. Borba, [Rio Madeira], 30 km along road from Borba to Rio Mapuri; disturbed terra firme forest on clayish soil, 24 Jun 1983 (fr), *C.A. Cid Ferreira 3912* (holotype: NY!; isotypes: INPA, RB-410311!, US-00842969!)

#### Description.

Liana or climbing shrub. Stems terete, striate, minutely ferruginous-pubescent, glabrescent, becoming dark brown to blackish with lines of reddish-brown lenticels when mature; cross section simple. Stipules subulate, 1–1.5 mm long, puberulent. Leaves partially tripinnate, with triangular outline; petiole narrowly winged or marginate, 1.6–2.5 cm long; rachis winged, 3.8–5 cm long, central ridge ciliate or glabrous, wings 1–2.5 mm wide; leaflets 6–15 × 4–8 mm, chartaceous, discolorous (abaxially lighter), glabrous or puberulent on both surfaces, sometimes abaxially glandular punctate, sessile and attenuate at base, obtuse, rounded, or truncate at apex, with dentate margins; distal leaflets rhomboid, with symmetrical base, lateral leaflets elliptic or obovate, with slightly asymmetrical base; venation craspedodromous, tertiary venation inconspicuous. Thyrses distal and paniculate or axillary and racemose, 15–32 cm long; peduncle 1.8–4 cm long; rachis 3.5–8.8 cm long, minutely appressed pubescent; bracts ca. 0.5 mm long, triangular, puberulent; cincinni 6- to 10-flowered, sessile; bracteoles ca. 0.2 mm long, similar to bracts; pedicels 0.5–0.7 mm long, articulate in lower third. Sepals 5, flavo-sericeous, concave, sub-coriaceous, ciliate, the outer sepals ovate, ca. 0.5 mm long; inner sepals obovate, 1–2 mm long; petals obovate, ca. 1.5 mm long; appendages hood-shaped, ca. 1 mm long, crest fleshy, yellow, bicorniculate in posterior appendages; nectary 4-lobed, pilose, the posterior lobes oblong-ovoid, truncate at apex, anterior lobes minute, torus pilose; sterile stamens with pilose filaments; gynoecium ca. 2 mm long, the ovary ellipsoid, sericeous-tomentose, style 0.1–0.5 mm long. Capsule depressed globose, unwinged, reddish, 5–10 × 7–12 mm, faintly 6-costate, crustaceous, flavo-puberulent, with slightly prominent vein network and a stipe 3–6 mm long; mesocarp 0.1–0.2 mm thick; endocarp glabrous. Seeds 1 or 2 per capsule, depressed globose, 5–7 × 7–9 mm, dark brown, pilose, with white, bilobed arillode in lower third; embryo depressed ovoid, cotyledons sub-straight.

#### Distribution and ecology.

Known only from the state of Amazonas, Brazil in non-flooded (*terra firme*) forest < 100 m elevation.

#### Phenology.

Collected in flower from May to July and in fruit in June.

#### Etymology.

The specific epithet honours Cid [Carlos A. Cid Ferreira], a functionary of INPA and prolific collector of Amazonian plants, who made the type collection.

#### Conservation status.

Known from five collections from lowland, non-flooded, moist forests NE and SE of Manaus, Amazonas, Brazil. Its known distribution has an extent of occurrence (EOO) of 22,000 km^2^. Since only a few collections are known of *P.cidii*, it is here treated as data deficient (DD) within IUCN guidelines.

#### Additional specimens examined.

BRAZIL. Amazonas, Rio Abacaxis, Terra Preta, terra firme forest, clayish soil, 4°22'S, 58°40'W, 5 Jul 1983 (fl), *Todzia et al. 2317* (INPA, MO, NY, RB, US); Município Presidente Figueiredo, Rebio Uatumã Grade do PPBio, 22 May 2007 (fl), *Zartman, et al. 7124* (INPA, US), Balbina, Rebio Uatumã, 1°00'S, 59°00'W, 15 Jul 2006 (fr), *Ribeiro et al. 2660* (INPA, US), Vila da Balbina, próximo a Ilha do Jacaré, 20 Jun 2006 (fr), *da Silva et al. 1207* (INPA, US).

#### Discussion.

*Paulliniacidii* is vegetatively similar to *P.bipinnata, P.filicifolia* and *P.hondurensis* by the characters mentioned in the diagnosis. Other species with similar fruits as those found in *P.cidii* include *P.carpopoda* Cambess. and *P.olivacea* Radlk. However, the leaves in *P.carpopoda* are 3-jugate (partially bipinnate), much larger and have entire margins (vs. smaller and serrate in *P.cidii*) while, in *P.olivacea*, they are 5-pinnate and the leaflets are much larger.

### Paullinia decorticans

Taxon classificationPlantaeSapindalesSapindaceae

Somner & Acev.-Rodr. sp. nov.

urn:lsid:ipni.org:names:77192861-1

Fig. 2

#### Diagnosis.

*Paulliniadecorticans* shares with *P.martinellii* Acev.-Rodr. & Somner the presence of bulbous axillary buds with large cataphylls and large overlapping bracts. However, *P.decorticans* differs from *P.martinellii* by the sericeous-lanate, glabrescent stems with defoliating bark (vs. tomentulose, glabrescent, without defoliating bark), the subulate 3–4 cm long stipules (vs. ovate to cordiform and clasping the stem, 3–5 cm long) and the depressed globose, sessile, puberulous fruits (vs. ellipsoid, long attenuate at the base and hirtellous).

**Figure 2.**
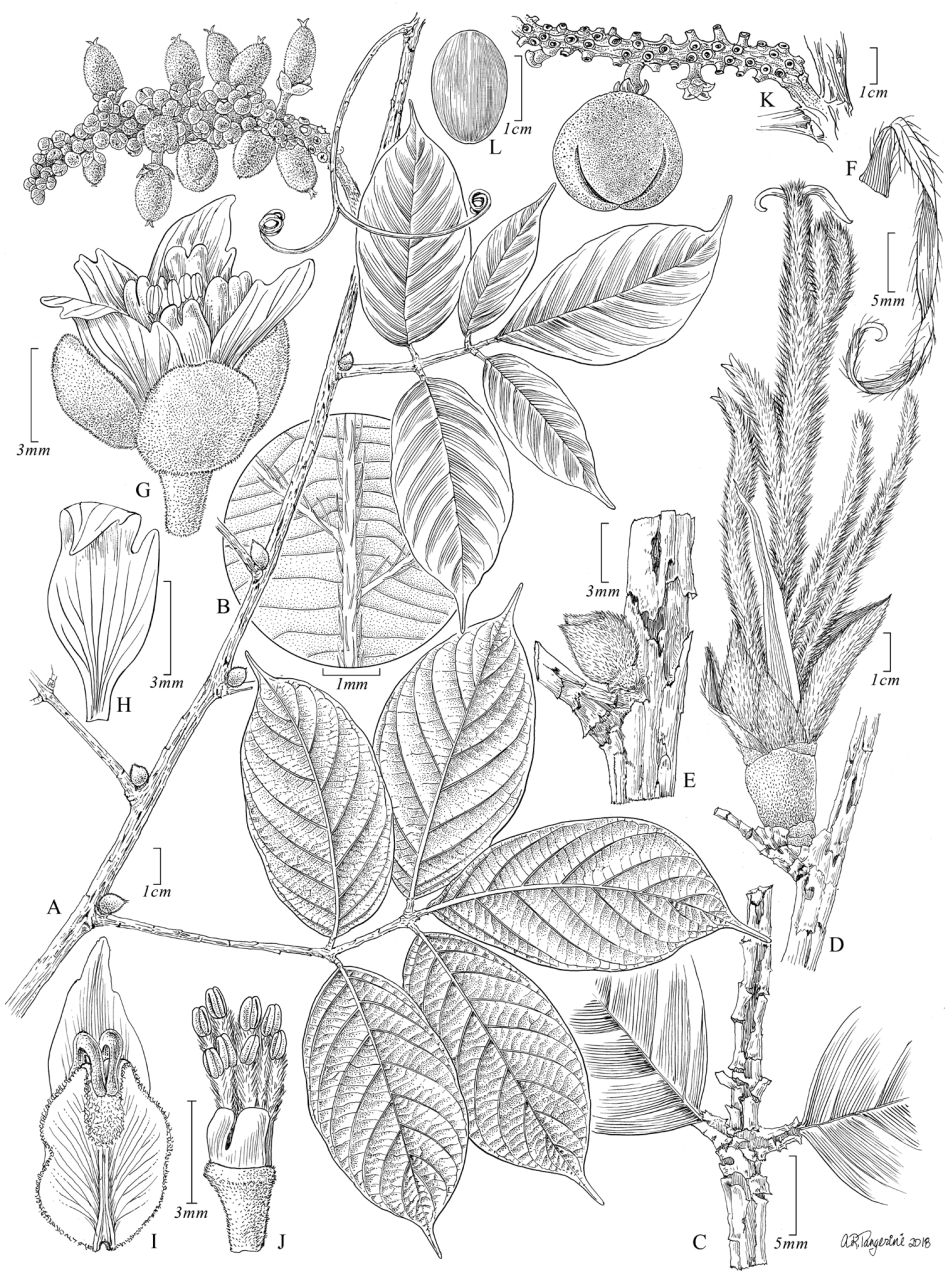
*Paulliniadecorticans*. **A** Fruiting branch **B** Detail of abaxial surface of leaflet **C** Insertion of two basal leaflets showing defoliating rachis and petiole **D** Axillary shoot with overlapping cataphylls **E** Bulbous, young axillary shoot covered by cataphylls **F** Stipule **G** Staminate flower, lateral view **H** Posterior petal, dorsal view **I** Posterior petal with appendage, frontal view **J** Staminate flower with removed perianth showing nectary lobes and stamens, posterior view **K** Axillary infructescence with a fruit **L** Seed, ventral view. **A, B, E, G–J** from *Burnham 1551* (US) **C** from *Cerón & Hurtado 4167* (US) **D** from *Vásquez & Jaramillo 17138* (MO) **F** from *Hurtado 2819* (MO) **K, L** from *Vásquez & Criollo 1804* (US).

#### Type.

ECUADOR. Napo; Yasuní National Park, 44–45 km along Maxus Petroleum Road, growing on trees along roadside, terra firme forest, 00°40'S, 76°23'W, 200–300 m elev., 18 Jan 1998 (fl, fr), *Burnham 1551* (holotype: US!; isotype: F!, QCA!)

#### Description.

Liana climbing on trees. Young stems sericeous-lanate, with beige, straight, trichomes, 5–8 mm long; mature stems to 3.3 cm diam., sub-terete, furrowed, glabrous, light brown, with lines of lenticels, producing abundant milky latex; bark thin, defoliating in small, rectangular plates; cross section simple, with solid medulla and lobed xylem. Axillary buds bulbous, ovoid, 8–25 mm long; cataphylls numerous, overlapping, acrescent, 5–10 × 1–3 cm, oblong, membranaceous, furfuraceous and sericeous. Stipules subulate 3–4 cm long, abaxially sparsely sericeous, early deciduous. Leaves pinnately 5- or 7-foliolate; petioles and rachis naked, sericeous when young, becoming glabrous and striate, with defoliating, thin epidermal layer; petioles 3–16(24.5) cm long; rachis 3–7.5(17) cm long; distal petiolules 5–15 mm long, lateral and basal petiolules 5–10 mm long; leaflets 9–14 (20) × 3–5 cm, chartaceous, elliptic, oblong or less often lanceolate or oblanceolate, adaxially glabrous, abaxially sparsely sericeous along primary and secondary veins, attenuate at base on distal leaflet, obtuse or rounded and asymmetrical on lateral leaflets, caudate at apex, with entire or undulate margins, venation brochidodromous, tertiary venation percurrent between secondary veins. Thyrses axillary, spicate, 5–14 cm long, axis robust, shortly flavo-tomentose, without tendrils; bracts 7–10 mm long, deltate-ovate, coriaceous, abaxially shortly flavo-tomentose, overlapping; cincinni 4- to 6-flowered, sessile; bracteoles 3–5 mm long, deltate-lanceolate, coriaceous, abaxially shortly flavo-tomentose; pedicels ca. 3 mm long, shortly flavo-tomentose, articulate in upper third. Sepals 5, shortly flavo-tomentose, concave, coriaceous, the outer sepals ovate, ca. 5.5 mm long, inner sepals suborbicular, 7–7.5 mm long, the two anterior sepals connate ca. ½ of their length; petals elliptic, ca. 12 mm long, atrolineate, acuminate at apex; appendages ca. 8 mm long, crest fleshy, bifurcate; nectary 2- or 4-lobed, glabrous, posterior lobes rectangular, brownish, ca. 2 mm long, anterior lobes deltate, ca. 1 mm long, or obsolete; torus flavo-tomentose; filaments densely lanate; gynoecium ca. 5.5 mm long, ovary trilobed-ellipsoid, densely ferruginous-tomentose. Capsule ellipsoid to globose-trilobed, unwinged, reddish, 3–4 × 2.2–3 cm, woody, puberulent or glabrous, sessile, apiculate at apex; pericarp ca. 8 mm thick. Seeds 1 to 3 per capsule, trigono-ellipsoid (with 2 flat sides), ca. 2.5 cm long, testa dark brown, dull, without arillode.

#### Distribution and ecology.

Known from NW Amazonia, in Ecuador and Peru in non-flooded, dense forest 140–400 m elevation.

#### Phenology.

Collected in flower in May, December and January and in fruit from December to January.

#### Etymology.

The specific epithet refers to the defoliating bark and epidermis of leaf axes present in the new species.

#### Conservation status.

Although *Paulliniadecorticans* is known from few collections, they come within an EOO of ca. 80,000 km^2^, which include the Yasuní National Park and densely forested areas within Loreto, Peru. Given the low level of threats associated with this vast region, this species is here treated as least concern (LC) within IUCN guidelines.

#### Additional specimens examined.

ECUADOR: Napo. Parque Nacional Yasuní, Pozo petrolero Daimi 2, primary humid, partly flooded forest along river, 00°55'S, 76°11'W, 200 m elev., 26 May–8 Jun 1988 (fl), *Cerón & Hurtado 4167* (US). Pastaza. Petroleum well Villano 2 de Arco, primary, humid forest, 01°25'S, 77°20'W, 400 m 1–18 Dec 1991 (fl), *Hurtado 2819* (MO), Mun. Puyo; Los Vencedores, Experimental Station ESPOCH, ca. 30 minutes by car, S of Puyo, premontane rainforest, 1°30'S, 77°56'W, 13 Dec 1995 (fl, fr), *Soerjato et al. 9392* (F). Orellana. Parque Nacional Yasuní, Parcela permanente de 50 ha del Proyecto “Dinamica del Bosque Yasuní a 1 km de la estación Científica del Yasuní, bosque muy húmedo, 0°41'00"S, 76°24'10"W, 230 m elev., 31 May 2006 (st), *Romero-Saltos et al., 2583* (QCA). PERU. Loreto. Quebrada Sucursari, (N side tributary of Río Napo), mature terra firme forest, 3°15'S, 72°55'W, 140 m elev., 14 Jun 1986 (st), *Gentry et al. 54367* (MO); Maynas. Explorama Lodge ½ way between Indiana and mouth of Napo river, mature forest on lateritic soil, 3°28'S, 72°50'W, 140 m elev., 5 Jan 1991 (st), *Gentry el al 72124* (MO); vicinity of Aguaytía, woods, 1 Jul 1967 (st), *Mathias & Taylor 5076* (F), Pto Alianza, Tamshiyacu creek, primary forest, 04°08'S, 72°55'W, 160 m elev., 28 May 1981 (fr), *Vásquez & Criollo 1804* (US), Explorama Inn, ca. 2 km W of Indiana on Rio Amazonas, well-drained forest on good soil, 3°30'S, 73°02'W, 130 m elev., 14 Feb 1987 (st), *Gentry et al. 55862* (F, MO), Iquitos, seasonally flooded, primary forest, 04°10'S, 73°30'W, 150–180 m elev., 6 Jul 1991 (st), *Vásquez & Jaramillo17138* (MO).

#### Vernacular name.

Equador. Pastaza: *Chunda* (fide *Hurtado 2819*), *Macote* (fide *Mathias 5076*).

#### Discussion.

*Paulliniadecorticans* differs from *P.martinellii*, the only other species of Paullinia with bulbous axillary buds with large cataphylls and large overlapping bracts by the features discussed in the diagnosis. *Paulliniadecorticans* is the only species within the genus recorded as having defoliating bark and defoliating leaf rachis epidermis.

### Paullinia fruticosa

Taxon classificationPlantaeSapindalesSapindaceae

Somner & Acev.-Rodr. sp. nov.

urn:lsid:ipni.org:names:77192862-1

Fig. 3

#### Diagnosis.

*Paulliniafruticosa* differs from *P.gigantea* Poepp. & Endl. and *P.killipii* Macbr., the only two other species of *Paullinia* with a combination of simple stems, 5-foliolate pinnate leaves, large foliaceous stipules and cauliflorous inflorescences, by its glabrous, bi-canaliculate, petioles 10.5–25 cm long, rachis 4.2–6.5 cm long; shrubby habit and the presence of unwinged fruits; these two congeners are lianas and their fruits are winged.

**Figure 3.**
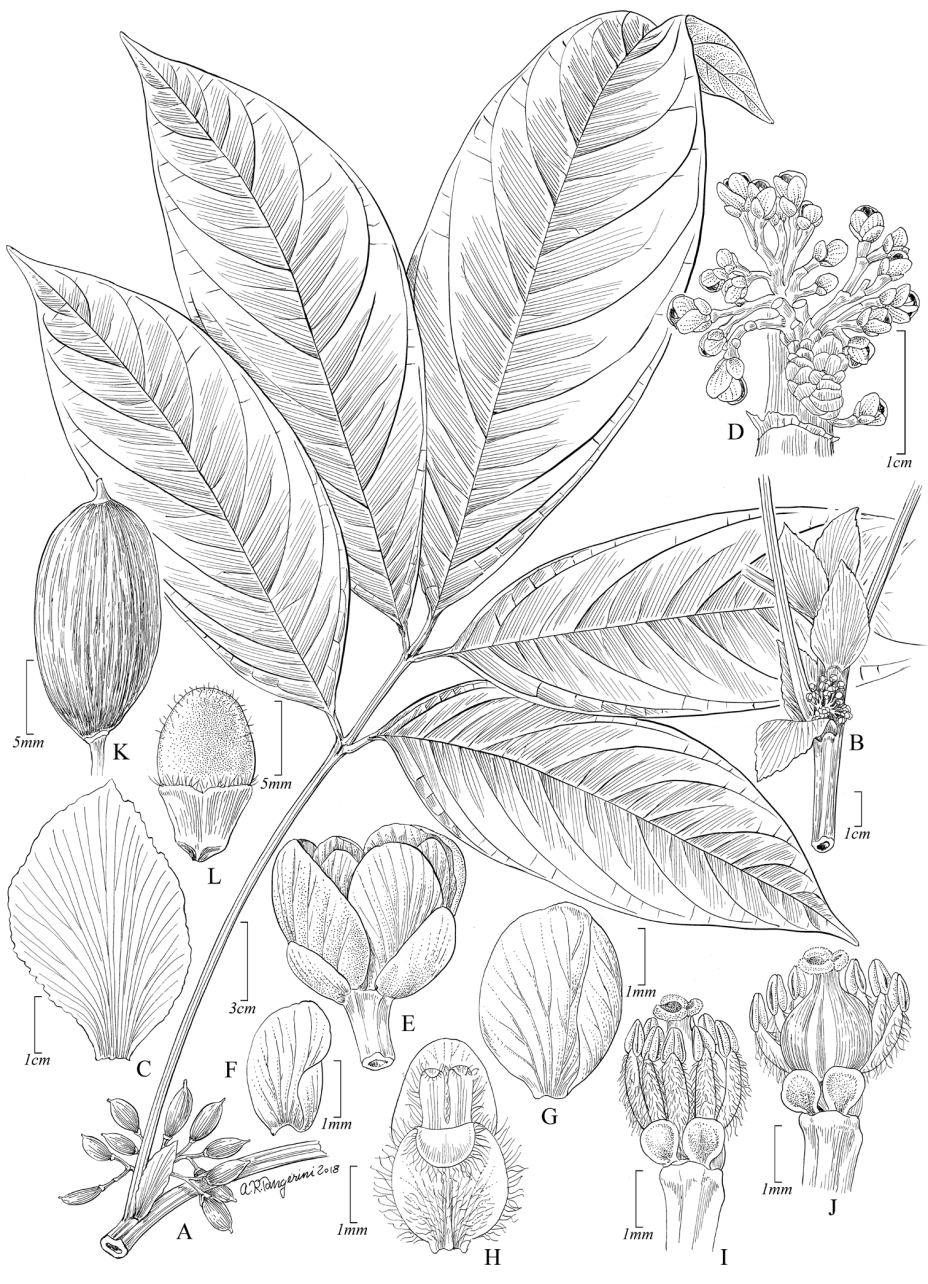
*Paulliniafruticosa*. **A** Fruiting branch **B** Detail of stems with stipules and axillary inflorescence **C** Posterior petal, dorsal view **D** Detail of inflorescence **E** Pistillate flower, lateral view **F** Outer sepal, frontal view **G** Posterior sepal, frontal view **H** Posterior petal with appendage, frontal view **I** Pistillate flower with removed perianth showing nectary lobes and staminodes, posterior view **J** Pistillate flower with removed perianth and some staminodes, showing nectary lobes and ovary, posterior view **K** Fruit **L** Seed, ventral view. **A** from *Foster et al 11693* (US) **B, D–L** from *Killip & Smith 27876* (US) **C** from *Klug 4200* (US).

#### Type.

PERU. Madre de Dios; Manú National Park, Río Sotileja, low floodplain forest, 400 m elev., 8 Oct 1986 (fr), *Foster et al. 11693* (holotype: US!; isotype: F!)

#### Description.

Erect shrub 1–2 m tall. Stems sulcate, glabrous, light green, sometimes fistulose, with white latex; cross-section simple. Stipules falcate-elliptic to rhombate, 2–3.1 × 1.5–2.2 cm, chartaceous, stramineous, glabrous, with numerous parallel, prominent veins. Leaves pinnately 5-foliolate; petiole and rachis naked, distal petiolules 1–1.5 cm long, lateral petiolules 0.3–0.7 cm long; leaflets 15–26 × 4.5–9.3 cm, chartaceous, glabrous, oblanceolate, elliptic, obovate or ovate, cuneate at base on distal leaflet, obtuse or acute and asymmetrical on lateral ones, obtusely acuminate or acuminate at apex, margins nearly entire or remote crenate-dentate, teeth with a submarginal gland, venation brochidodromous or mixed craspedodromous, abaxially prominent, especially the midvein, tertiary venation reticulate. Thyrses axillary, glomerate, axes glabrous, ca. 2 cm long; bracts ca. 1 mm long, deltate, glabrous; cincinni 5- to 7-flowered; peduncle 4–8 mm long; bracteoles ca. 0.5 mm long, triangular, ciliate; pedicels 5–6.5 mm long, articulate near the base. Sepals 5, glabrous, yellowish, membranaceous, ciliate, the outer sepals ovate, 2–2.5 mm long, inner sepals obovate, 3–3.5 mm long; petals white, elliptic, ca. 3.5 mm long; appendage ca. 3mm long, crest of posterior appendage with 2 corniform projections; nectary 4-lobed, posterior lobes ovate, obtuse or truncate at apex, anterior lobes smaller, pilose; torus glabrous; filaments pubescent, 2–4 mm long, flat, anthers glabrous; gynoecium ca. 2 mm long, the ovary ovoid, ca. 1 mm long, sparsely villose. Capsule ellipsoid, unwinged, 1–1.8 × ca. 0.7 cm, coriaceous, glabrous, densely parallel-veined, sessile, apiculate at apex; mesocarp ca. 0.5 mm thick; endocarp lanose. Seed one per capsule, ellipsoid, 0.9–1.5 cm long, pubescent, with bilobed sarcotesta on lower ½; embryo, elliptic, ca. 0.8 × 0.5 cm, abaxial cotyledon curve, adaxial cotyledon biplicate.

#### Distribution and ecology.

Known from SW Amazonia, in Peru and the state of Acre in Brazil, in non-flooded, dense forest 100–500 m elevation.

#### Phenology.

Collected in flower from August to November and in fruit from October to December and June.

#### Etymology.

The specific epithet refers to the shrubby habit of the new species.

#### Conservation status.

Known from ten collections within an EOO of 150,000 km^2^, in moist forest understorey in the south-western Amazon region. Although this species is known only from few collections, its conservation status is here treated as least concern (LC) due to its occurrence within Manú National Park in Peru.

#### Additional specimens examined.

BRAZIL. Acre. Mun. Mâncio Lima; Upper Rio Moa, base of Serra Azul, ca. 07°29'S, 73°39'W, 12 Oct 1986 (fr), *Campbell et al. 8947* (NY). PERU: Loreto; Puerto Arturo, lower Rio Huallaga, below Yurimaguas, ca. 135 m elev., dense forest, 24–25 Aug 1929 (fl), *Killip & A.C. Smith 27876* (US), Santa Rosa, ca. 135 m elev., dense forest, 1–5 Sep 1929 (fl), *Killip & A.C. Smith 28741* (US), *28876* (F, US). Madre de Dios; Manú, Manú settlement in forest, 4 Aug 1973 (fl), *Foster 2469* (F), Parque Nacional Manú, Rio Sotileja, steep forested hills along stream, 11°40'S, 71°55'W, 400–500 m elev., 2 Oct 1986 (fl), *Foster et al. 11579* (F). San Martín, Juanjui, upper Río Huallaga, 400–800 m elev., forest, Dec 1935 (fr), *Klug 4200* (F, US). Ucayali, Trail from Quebrada Shesha (tributary of Río Abujao) to base of Cerro las Cachoeiras, ca. 70 km NE of Tucallpa, 08°02'S, 73°55'W, 300–400 m elev., 24 Jun 1987 (fr), *Gentry & Diaz 58491* (MO, QCA); Coronel Portillo, Callería, Cuenca del Río Utiquinia, Quebrada Espjoyacu, afluente de la Quebrada Manuela, primary forest, 07°56.67'S, 73°53.61'W, 300 m elev., 8 Nov 2003 (fl, fr), *Graham 2636* (US).

#### Discussion.

*Paulliniafruticosa* is the only species in the genus that is consistently described in collection labels as a shrub. Other species of *Paullinia* (e.g. *P. cuneata Radlk.* and *P.dasystachya*) may present a shrubby habit but only during early stages but later developing into lianas. In addition, *Paulliniakillipii*. was originally described as a shrub, based on mixed collections including *Killip & A.C. Smith 27876* and *28876*, which are referable to *P.fruticosa*. As seen in several collections, *P.killipii* develops as a liana that grows into the forest’s canopy. These three species also differ from *P.fruticosa* by the presence of winged fruits.

### Paullinia hondurensis

Taxon classificationPlantaeSapindalesSapindaceae

Acev.-Rodr. & Somner sp. nov.

urn:lsid:ipni.org:names:77192863-1

Fig. 4

#### Diagnosis.

*Paulliniahondurensis* has partially tripinnate leaves, a character that is shared only with *P.bipinnata* Poir., *P.cidii* Somner & Acev.-Rodr. and *P.filicifolia* Cuatrec. *Paulliniahondurensis* can be distinguished from *P.bipinnata* by the glabrous stems, inflorescence axes and calyx (vs. tomentose), as well as by the glabrous and 2–2.8 cm long capsules (vs. pubescent, 1 cm long). *Paulliniahondurensis* can be distinguished from *P.cidii* by its deltate, 2–3 mm long stipules, the nearly terete petioles and the 3-winged, trilobed-turbinate capsules (vs. subulate, 1–1.5 mm long stipules, winged or marginate petioles and unwinged depressed globose capsules). *Paulliniahondurensis* differs from *P.filicifolia* by the glabrescent stems and foliage (vs. pubescent), by the distal leaflets with obtuse apex (vs. acuminate) and by the coriaceous, obtriangular, glabrous capsules with straight wings (vs. chartaceous, ellipsoid, pubescent capsules with revolute wings).

**Figure 4.**
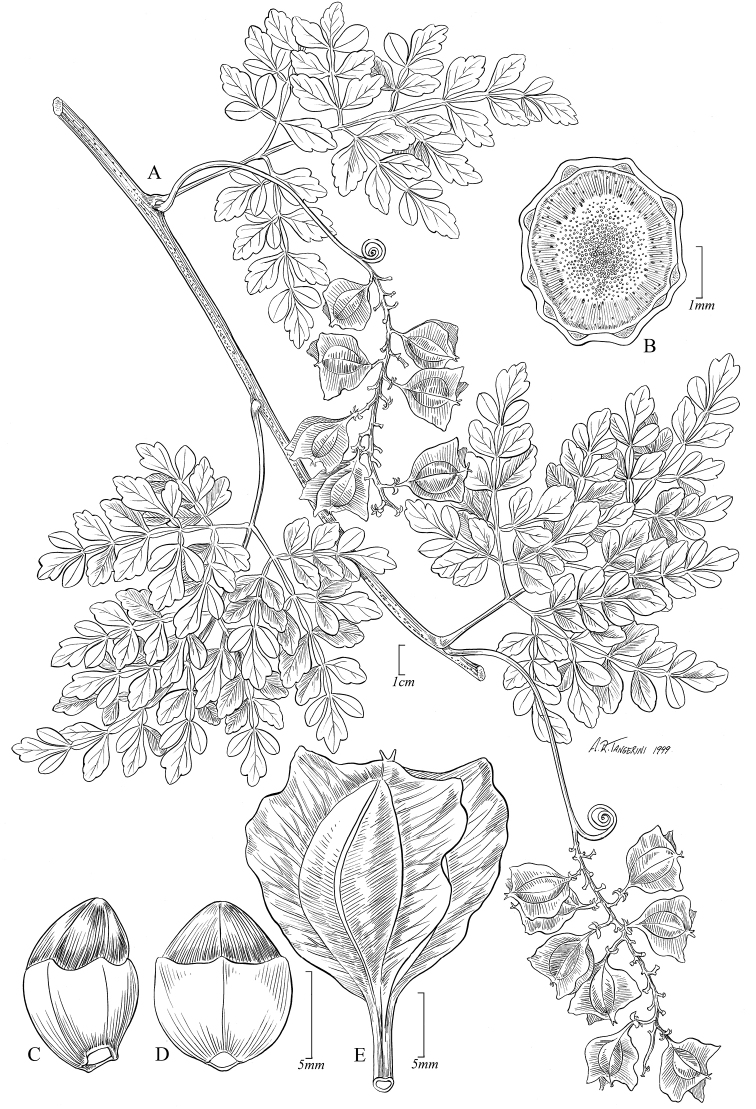
*Paulliniahondurensis*. **A** Fruiting branch **B** Detail of cross section of stem **C** Seed, lateral view **D** Seed, frontal view **E** Fruit. All from *Thomas et al. 139* (TEFH).

#### Type.

HONDURAS. Dept. F. Morazán; [National Park La Tigra], humid and cloud forests, Rancho Quemado, Montaña La Tigra, 2000 m elev., 10 Dec 1958 (fr), *A. Molina R. 8767* (holotype: US!; isotypes: EAP, MO).

#### Description.

Vine 4–6 m long. Stems nearly terete, striate, reddish-brown, minutely pubescent when young, glabrescent and with lines of minute lenticels when mature; cross section simple. Stipules deltate, 2–3 mm long. Leaves partially tripinnate, with triangular outline; petioles 2.2–4.5 cm long, nearly terete, adaxially bi-canaliculate, with a central pubescent ridge; rachis 6–10 cm long, marginate or with wings 1–2.5 mm wide, revolute, pubescent along central ridge; leaflets 1.6–2 × 0.6–1.3 cm, subcoriaceous, discolorous, glabrous, attenuate at base, acute or obtuse and sometimes mucronate at apex, margins serrate near the apex, distal leaflets rhomboid, lateral and basal leaflet elliptic, obovate or ovate, slightly asymmetrical at base; venation mixed craspedodromous, adaxially with prominent pubescent midvein, abaxially with midvein and secondary veins prominent and hair domatia on lower secondary axils; tertiary venation reticulate. Thyrses axillary, racemose, 4–9 cm long, with pubescent axes; bracts ca. 2 mm long subulate; cincinni shortly stipitate, 3- or 4-flowered; pedicels 2–3 mm long, articulate at middle. Sepals 5, concave, chartaceous, ciliate, the outer sepals ovate 2.5–2.7 × 1.8–1.9 mm, inner sepals sub-orbicular or obovate, 3.4–3.5 × 2.6–3.2 mm; petals oblong, unguiculate, ca. 4 × 2 mm, with glandular trichomes on adaxial surface; appendages ca. 3 mm long, crest emarginate; nectary 4-lobed, puberulent, the posterior lobes ca. 1 mm long, ovate, anterior lobes ca. 0.6 mm, elliptic; stamens unequal, free to base, 2–2.7 mm long, filament pubescent; ovary glabrous. Capsule trilobed-turbinate, 3-winged, red, 2–2.8 × 1.5–2.3 cm, coriaceous, prominently veined, with subglobose coccus, glabrous, truncate and apiculate at apex, wings dorsal-apical (on upper half portion of capsule), 4–7 mm wide, cuneate at base and with stipe 2.5–5 mm long; mesocarp ca. 0.5 mm thick; endocarp yellowish-brown tomentose. Seeds 1–3 per capsule, trigonous-obovoid, ca. 1 × 0.8 cm long, dark brown, glabrous, shiny, with bilobed arillode in lower half; embryo ellipsoid; abaxial cotyledon curve, adaxial cotyledon biplicate.

#### Distribution and ecology.

In moist or cloud forest between 1900–2000 m elevation.

#### Phenology.

Known to flower in July and to fruit in December and January.

#### Etymology.

The specific epithet refers to the country where the species was first collected.

#### Conservation status.

Known only from four localities from montane cloud forests (1990–2000 m elevation) in Guatemala and Honduras, with an approximate EOO of 11,000 km^2^. The type collection indicates that the species was frequent in the area, however, due to the small area of occurrence and the lack of recent collections, the species is here treated as vulnerable (VU) within IUCN guidelines.

#### Additional specimens examined.

GUATEMALA. Prov. Alta Verapaz. Mun. San Juan Chamelco. Montaña Caquipec, from Caquipec to Chicacnab I, 15°22'49"N, 90°10'59"W, ca. 1900 m, secondary forest and primary cloud forest, 9 Sep 1999 (st), *Förther 10489* (US). HONDURAS. Dept. Lempira. Dense, mixed cloud forest on the east slopes of Quebrada Naranja, 10 km SE of Gracias, Celaque National Park, 14°33'N, 88°40'W, 1950 m, 29 Jan 1992 (fr), *Thomas et al. 139* (EAP, HEH, MO, TEFH). Dept. F. Morazán, National Park La Tigra, SW of San Juancito, dense, cloud, montane forest, 2000 m, 14 Jul 1961 (fl), *A. Molina R. 10120* (US).

#### Discussion.

We have not been able to infer the phylogenetic relationship of the new species with any other species in the genus due to the lack of adequate quality genome material. The suggested relationships with *P.bipinnata, P.cidii* and *P.filicifolia* are based on the overall morphological similarity discussed in the diagnosis. The current infrageneric classification of *Paullinia* based on fruit morphology (Radlkofer 1895, 1934) would place the new species in the proximity of *P.tricornis* Radlk., as both species have winged, trilobed-turbinate, prominent veined capsules with subglobose cocci. However, *P.hondurensis* clearly differs from *P.tricornis* by the partially tripinnate leaves (vs. 5-pinnate).

*Paulliniahondurensis* is easily confused with sterile collections of *Serjaniarhachitera* Radlk., which has tripinnate leaves with winged rachis and an overlapping distribution. However, sterile *S.rachiptera* can be distinguished *from P.hondurensis* by the sulcate stems that lack lenticels and leaflets that lack hair domatia.

### Paullinia martinellii

Taxon classificationPlantaeSapindalesSapindaceae

Acev.-Rodr. & Somner sp. nov.

urn:lsid:ipni.org:names:77192864-1

Fig. 5

#### Diagnosis.

*Paulliniamartinellii* is unique within the genus by its large (3–5 cm long), membranaceous, clasping stipules and overlapping cataphylls and by the large (ca. 1 cm long), overlapping, boat-shaped bracts, resembling no other species of *Paullinia*.

#### Type.

BRAZIL. Pará; Mun. Oriximiná, Rio Caxipacoró, km 72 on the road north of Cachoeira Porteira, along disturbed roadside, 30 Jun 1980 (fl, fr), *Davidson & Martinelli 10661* (holotype US!; isotypes INPA!, NY!).

**Figure 5.**
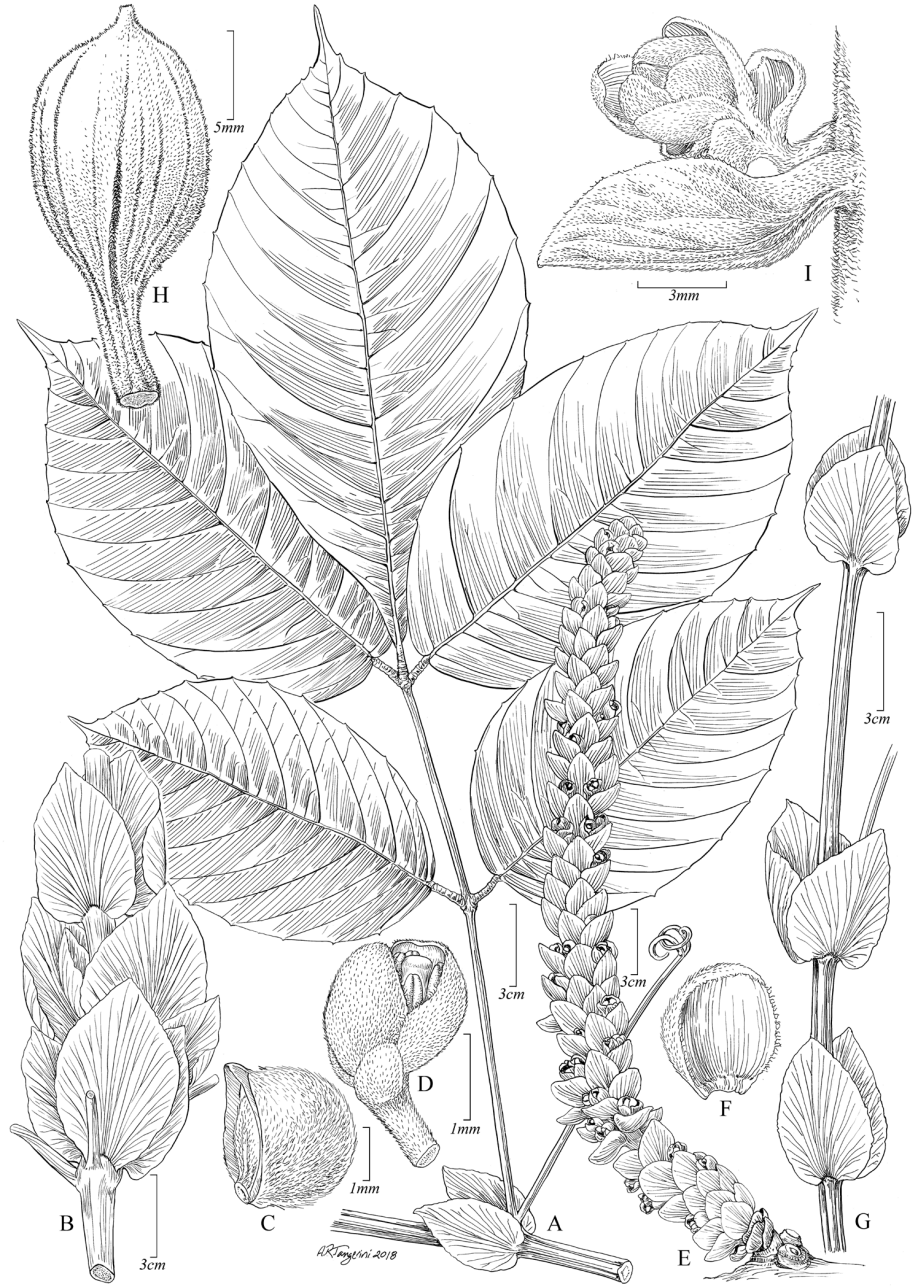
*Paulliniamartinellii*. **A** Branch fragment with leaf, stipules and tendrils **B** Branch with cataphylls **C** Flower bud covered by bracts and bracteoles **D** Immature flower **E** Inflorescence **F** Posterior sepal, frontal view **G** Branch showing stipules **H** Immature fruit **I** Immature cincinnus with overlapping bracts and bracteoles. **A–D, F, H** from *Davidson & Martinelli 10661* (US) **E, G** from *Martinelli & Davidson 7158* (RB) **I** from *Cid et al. 1223* (INPA).

#### Description.

Liana. Stems furrowed and tomentulose when young, becoming glabrous, terete and lenticellate with age; cross-section simple; xylem with wide rays and phloem wedges. Tendrils similar to those in other *Paullinia* but not associated with inflorescences. Cataphylls overlapping, similar to stipules in size and texture, produced at base of lateral branches. Stipules foliaceous, membranaceous, ovate to cordiform, clasping the stem, 3–5 × 2–4 cm, puberulous, conspicuously veined, with undulate margins. Leaves pinnately 5-foliolate; petiole and rachis nearly terete, adaxially furrowed, tomentose or tomentulose; petiole 14.5–16.5 cm long, pulvinate at base; rachis 6–8.5 cm long; petiolules pulvinate, tomentulose, distal petiolule ca. 1 cm long, lateral petiolules 2–5 mm long; leaflets membranaceous with linear punctations, glabrous except veins tomentulose on adaxial surface and puberulent on abaxial surface, acuminate at apex, margins dentate or subentire, with secondary veins projecting as glandular teeth; venation craspedodromous, abaxially prominent; tertiary venation reticulate-percurrent between the secondary veins; distal leaflet 24–25 × 12–15.5 cm, broadly elliptic to obovate, attenuate at base; lateral leaflets 15–18 × 9–11 cm, oblong to ovate, rounded or sub-cordate at base. Thyrses cauliflorous, lacking tendrils, in fascicles of 5–7; axes robust, woody, 20–34 cm long, ferruginous-tomentose; bracts ovate concave, crustose, 8–10 × 6–8 mm, minutely sericeous; cincinni 2- or 3-flowered, sessile; bracteoles broadly elliptic, concave, tomentulose, ca. 5 mm long, covering the cincinnus; pedicels articulate at base. Sepals 4, sericeous tomentose, crustose, the outer sepals ovate, ca. 5 mm long, inner sepals broadly elliptic, concave, 5.5–6 mm long, anterior sepal emarginate; petals obovate, ca. 6.5 mm long, abaxially covered with glandular trichomes; appendages hood-shaped, ca. 5 mm long, with fleshy, yellow, corniform crests, two in posterior appendages, one in anterior appendages; nectary 4-lobed, posterior lobes rectangular with emarginate or truncate apex, anterior lobes triangular; torus villose; filaments connate at base, lanate throughout, 2–3 mm long; anthers glabrous. Capsule (immature) ellipsoid, unwinged, orange, ferruginous-hirtellous, long-attenuate at base, and obtuse and apiculate at apex.

#### Distribution and ecology.

Terra firme forest at elevations below 100 m.

#### Phenology.

Old flowers and young fruits known from June–July.

#### Etymology.

The specific epithet honours Dr. Gustavo Martinelli, eminent Brazilian botanist at Rio de Janeiro Botanical Garden, who made the first collection of this species.

#### Conservation status.

Known from three collections from two localities with an EOO of ca. 3,000 km^2^ in the Municipality of Oriximiná in the state of Pará, Brazil. The new species seems to be rare and, because of its small known EOO, it is here treated as vulnerable (VU) within IUCN guidelines.

#### Additional specimens examined.

BRAZIL. Pará; Mun. Oriximiná, Estrada da Cachoeira Porteira km 72, próximo ao igarapé Caxicoporó, margem esquerda, mata firme, solo argiloso, 1 Jul 1980 (fl), *Cid Ferreira et al. 1223* (INPA), Rio Cachorro, Cachoeira de Varador, terra firme forest, 90 m elev., 22 Jun 1980 (fl, fr), *Martinelli & Davidson 7158* (MG, NY, RB).

#### Discussion.

*Paulliniamartinellii* is the only species within the genus known to have large (3–5 cm long), membranaceous, clasping stipules and large (ca. 1 cm long), overlapping, boat-shaped bracts. Its position within the genus has not been ascertained as no adequate material is available for carpological or molecular analyses.

### Paullinia wurdackii

Taxon classificationPlantaeSapindalesSapindaceae

Acev.-Rodr. & Somner sp. nov.

urn:lsid:ipni.org:names:77192865-1

Figs 6 , 7

#### Diagnosis

. *Paulliniawurdackii* differs from *P.ingifolia* Juss. by the lanate or lanate-tomentose stems and inflorescence axes (vs. tomentose or tomentulose), the oblong-lanceolate, ovate or deltate bracts 9–15 mm long that enclose the cincinni (vs. deltate, 1–2.5 mm long, not enclosing the cincinni), the ovate-lanceolate bracteoles 4–5 mm long (vs. deltate, ca. 1 mm long) and the sessile, depressed-globose capsules (vs. long stipitate, globose or trigonous-globose).

#### Type.

ECUADOR. Orellana; Yasuní Forest Reserve, 1 km W of PUCE Scientific Station, moist forest along road, 0°41.956'S, 76°28.075'W, 250–300 m elev., 5 Jul 1995 (fl♀), *Acevedo-Rodríguez & J.A. Cedeño 7654* (holotype US!, isotype QCA).

**Figure 6.**
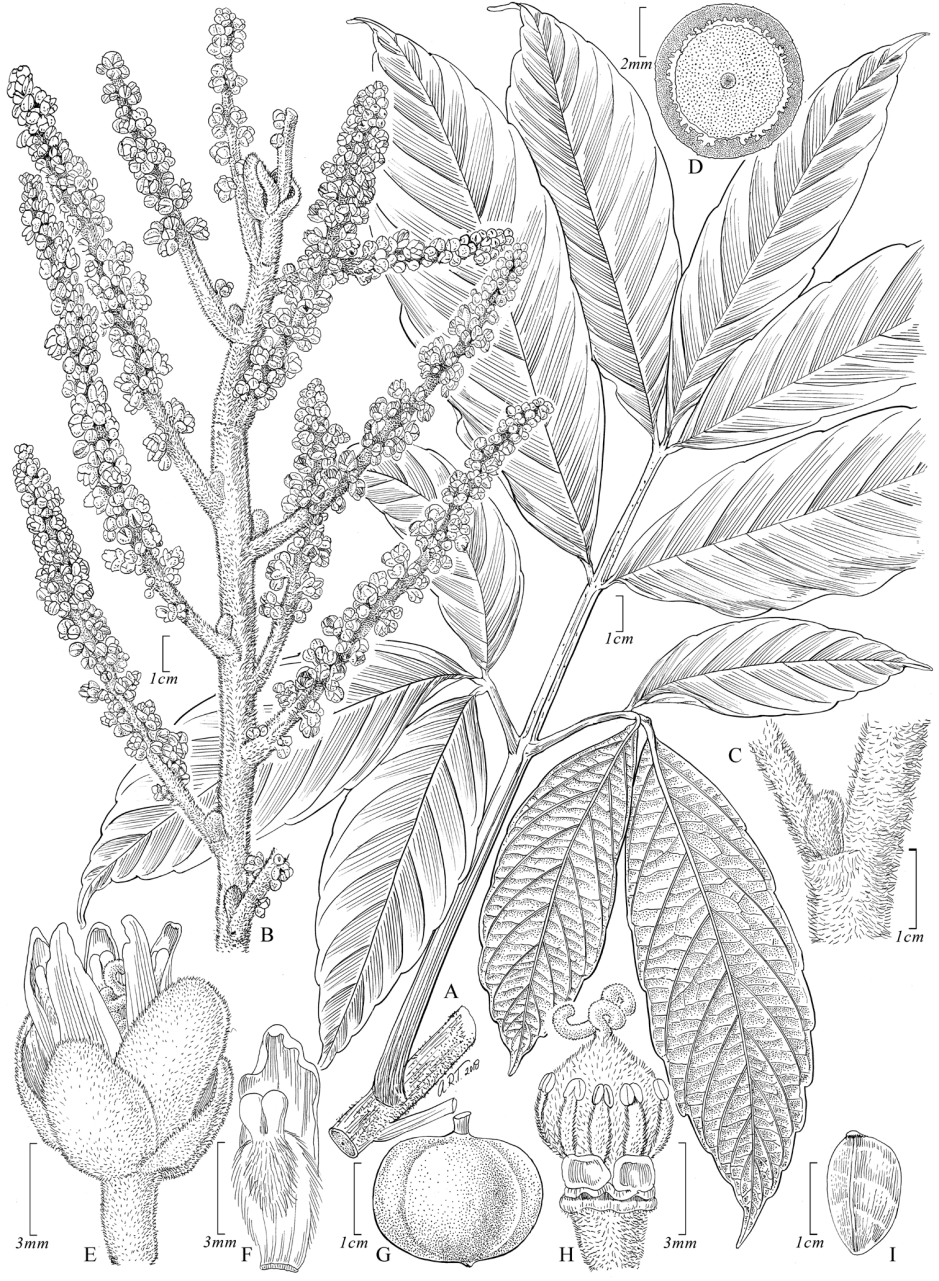
*Paulliniawurdackii*. **A** Leaf with stem fragment **B** Inflorescence **C** Bulbous, axillary bud **D** Cross section of stem **E** Pistillate flower, lateral view **F** Posterior petal with appendage, frontal view **G** Fruit **H** Flower with removed perianth showing nectary lobes, staminodes, and gynoecium **I** Seed, ventral view. **A** from *Croat 58698* (MO) **B–F, H** from *Acevedo-Rodríguez & Cedeño 7654* (US) **G, I** from *Pitman 602* (US).

#### Description.

Liana 20–25 m long. Stem terete, ferruginous lanate or lanate-tomentose, becoming glabrous and lenticellate with age, producing abundant milky sap; cross section simple, up to 10 cm diam. Stipules early deciduous, leaving a scar 7–10 mm wide. Leaves imparipinnate with 1 basal pair of trifoliolate pinnae and 2 or 3 pairs of simple lateral leaflets (thus with 11 or 13 ultimate leaflets); petiole and rachis winged; petiole 10.5–23.5 cm long, wings 0.4–0.8 cm wide; rachis 7–14 cm long, wings 0.4–0.7 cm wide, puberulent; lower pair of trifoliolate pinnae with winged petiolules 1.4–1.8 cm long; leaflets 13.5–16 × 3.8–4.5 cm, coriaceous, oblong, oblong-elliptic or less often oblanceolate, discolorous, abaxially papillate; cuneate at base on distal leaflet, acute, obtuse, attenuate or sometimes asymmetrical on lateral leaflets, obtusely acuminate to caudate at apex with margins remotely serrate on distal third to half of the blade, teeth with a sub-marginal gland-like callosity; venation craspedodromous, abaxially prominent with scattered setose trichomes, without domatia, tertiary venation reticulate-percurrent between secondary veins. Thyrses terminal, paniculate, 24.5–27 cm long; axes robust, ferruginous lanate or lanate-tomentose; branches 8–16 cm long, with axillary bulbous buds; cataphylls oblong, concave, 13–37 × 8–17 mm, ferruginous sericeous-lanate, deciduous, leaving a scar 5–12 mm wide; bracts 9–15 mm long, oblong-lanceolate, ovate or deltate, flavo sericeous, enclosing the cincinni, which are clothed by overlapping bracteoles, similar in shape, texture and indument to the bracts, but smaller (4–5 mm long), both bracts and bracteoles early deciduous; cincinni 5- to 7-flowered, sessile or sub-sessile; pedicels 2–3 mm long, articulate at middle. Calyx light green when fresh, albo-tomentose when dry, sepals 4, concave, coriaceous, outer sepals ovate, 4–5.5 mm long, inner sepals oblong-ovate, 4.5–7 mm long, the anterior sepal emarginate; petals white to cream, oblong, 4.5–7 mm long, papillate, appendages hood-shaped, 4–5 mm long, crest of posterior appendages emarginate; nectary 4-lobed, the posterior lobes ca. 1.5 mm long, oblong, emarginate at apex, anterior lobes oblong, smaller than the posterior; filaments lanate, 4.5–7 mm long, connate at the very base; gynoecium 3–3.5 mm long; ovary trilobed-ovoid, flavo sericeous-tomentose. Capsule depressed-globose, unwinged, 2–2.3 × 1.8–2 cm, 3-costate, woody, ferruginous sericeous-tomentose, sessile, apiculate at apex; mesocarp 2.5–3 mm thick; endocarp sericeous-tomentose. Seeds oblong-obovate, trigonous in transverse section, entirely sarcotestal, ca. 1.5 cm long.

#### Distribution and ecology.

Western Amazonia, in moist forests between 250–375 m elevation.

#### Phenology.

Flowering from October to April and known to fruit in July.

#### Etymology.

The specific epithet honours the late Dr. John J. Wurdack from the Smithsonian Institution, Curator of Melastomataceae and assiduous collector of South American plants, who made one of the first collections of this species.

#### Conservation status.

Known from an EOO of ca. 150,000 km^2^ in western Amazonia on Ecuadorian and Peruvian territories. The new species is known only from 5 collections from this vast area, which seems to indicate that the new species is extremely rare. However, because the species occurs in a National Park in Ecuador and in vastly forested areas of Peru with no immediate threats to the ecosystem, the new species is here treated as least concern (LC) within IUCN guidelines.

#### Additional specimens examined.

ECUADOR. Orellana. Aguarico, Reserva Etnica Huaorani, maxus road and pipeline construction Project, km 100–102, moist primary forest on red soils & undulating hills, 0°56'S, 76°13'W, 250 m elev., 18 Jul 1994 (fr), *Pitman 602* (MO, QCNE, US); Sucumbíos, 9.3 km E of Lumbaqui, ca. 0°06'N, 77°16'W, 375 m elev., 29 Apr 1984 (fl), *Croat 58698* (MO, NY, QCA, US). PERU. Amazonas. Pongo de Manseriche, on high land, without date (fl), *Tessmann 3889* (NY); Provincia Bagua, forested ridge on right bank of Río Santiago, 3–4 km above mouth, 300–350 m elev., 29 Oct 1962 (fl ♂, ♀, fr), *J.J. Wurdack 2476* (US).

#### Discussion.

*Paulliniawurdackii* is vegetatively similar to *P.ingifolia*. as both species are robust lianas with imparipinnate leaves with trifoliolate lower pinnae and winged rachides and petioles; oblong, elliptic or oblanceolate leaflets with caudate apices; large deciduous stipules; and unwinged, woody fruits. *Paulliniawurdackii* however, is easily recognised by its lanate or lanate-tomentose stems and inflorescence axes, the large bracts and overlapping bracteoles that enclose the cincinni and the sessile fruits. While *P.ingifolia* has a wide distribution that ranges from Costa Rica south to Bolivia, *P.wurdackii* is restricted to the NW region of the Amazon basin.

**Figure 7.**
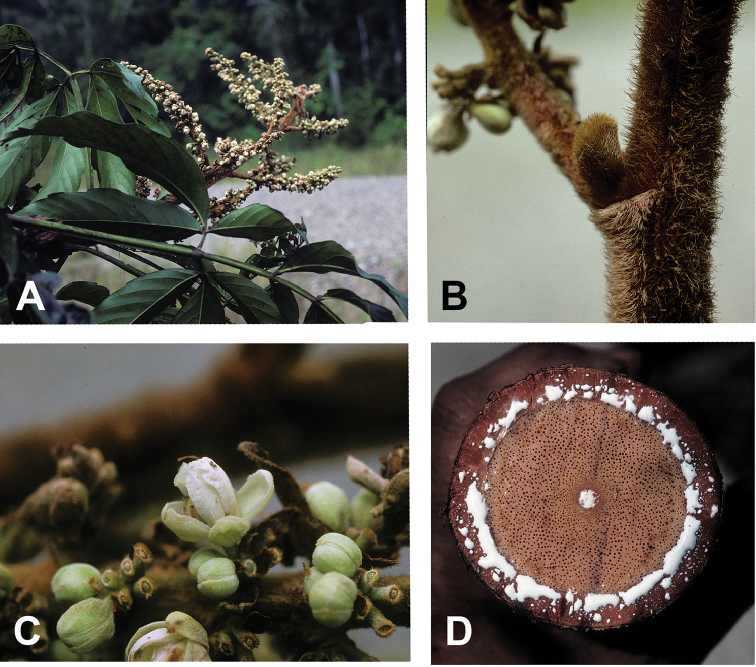
*Paulliniawurdackii*. **A** Flowering branch **B** Inflorescence axis with lanate indument and bulbous axillary bud covered by cataphylls **C** Detail of inflorescence showing sessile cincinni and few remnant bracts **D** Cross section of stem showing regular anatomy and abundant milky latex emanating from the phloem and medulla. Photos by P. Acevedo, based on *Acevedo-Rodríguez & Cedeño 7654*.

## Supplementary Material

XML Treatment for Paullinia cidii

XML Treatment for Paullinia decorticans

XML Treatment for Paullinia fruticosa

XML Treatment for Paullinia hondurensis

XML Treatment for Paullinia martinellii

XML Treatment for Paullinia wurdackii
